# Western Ontario Veterinary Medical Association

**Published:** 1897-02

**Authors:** Jacob Wagner

**Affiliations:** Secretary


Secretary.

### WESTERN ONTARIO VETERINARY MEDICAL ASSOCIATION.

THE semiannual meeting of this Association was held in the City Hall, Stratford, December 29, 1896, President William Gibb, of St. Mary's, occupying the chair. There was a fairly large attendance of members present from different parts of the peninsula.

After reading of the minutes of the last meeting an address was delivered by the President, followed by the report of the Legislative Committee. Papers were read by Dr. Campbell, Hawkesville, on "Lymphangitis;" Dr. George H. Gibb, Seaforth, on "Removal of a Portion of the Spinal Column of a Cow;" Dr. William Gibb, St. Mary's, on "Value of Action and Position as Indications of Lameness in the Horse, and the Diagnosis of its Position." Other interesting and instructive papers were read by Dr. Bowker, Woodstock; Dr. Orr, Baden; Dr. Wilson, Wingham; Dr. Clark, Goderich; Dr. Engle, Milverton; Dr. Hodgins, Stratford; Dr. Eckert, Sebringville. Dr. William Gibb was re-elected a member of the Board of Directors. The fee and annual dues were reduced and several new members were proposed and accepted.

JACOB WAGNER,
Secretary.

